# CYP 2D6 Binding Affinity Predictions Using Multiple Ligand and Protein Conformations

**DOI:** 10.3390/ijms141224514

**Published:** 2013-12-17

**Authors:** Lovorka Perić-Hassler, Eva Stjernschantz, Chris Oostenbrink, Daan P. Geerke

**Affiliations:** 1 AIMMS Division of Molecular Toxicology, Department of Chemistry and Pharmaceutical Sciences, Faculty of Sciences, VU University Amsterdam, De Boelelaan 1083, 1081 HV Amsterdam, The Netherlands; E-Mail: lovorka.peric@gmail.com; 2 Institute of Molecular Modeling and Simulation, University of Natural Resources and Life Sciences, Muthgasse 18, 1190 Vienna, Austria; E-Mail: chris.oostenbrink@boku.ac.at

**Keywords:** binding free energies, LIE method, CYP 2D6, thiourea ligands

## Abstract

Because of the large flexibility and malleability of Cytochrome P450 enzymes (CYPs), *in silico* prediction of CYP binding affinities to drugs and other xenobiotic compounds is a true challenge. In the current work, we use an iterative linear interaction energy (LIE) approach to compute CYP binding affinities from molecular dynamics (MD) simulation. In order to improve sampling of conformational space, we combine results from simulations starting with different relevant protein-ligand geometries. For calculated binding free energies of a set of thiourea compounds binding to the flexible CYP 2D6 isoform, improved correlation with experiment was obtained by combining results ofMDruns starting from distinct protein conformations and ligand-binding orientations. This accuracy was obtained from relatively short MD simulations, which makes our approach computationally attractive for automated calculations of ligand-binding affinities to flexible proteins such as CYPs.

## 1. Introduction

The prediction of ligand-protein binding affinities or binding *free energies* is not only a major challenge in structure-based drug design, but also for *in silico* studies on metabolism of xenobiotics. Different (drug-)metabolizing enzymes may catalyze the formation of different products. Therefore, predicting the affinity and selectivity towards these enzymes can be an important step in (drug-)metabolite prediction. Although there are many approaches available to calculate protein binding free energies [1,2], most of them lack either accuracy [3–6] or computational efficiency [7,8]. In the past, Å qvist and co-workers [9] have introduced the linear interaction energy (LIE) approach as a relatively fast method for binding affinity computation with satisfactory accuracy [5,10].

LIE is based on linear response theory and determines ligand solvation free energies from molecular dynamics (MD) simulations at only two states, *i.e*., when bound to the protein, and when being unbound (freely solvated) in water. The simplicity of this method makes it appealing to use. However, its accuracy or efficiency may be restricted by the required conformational sampling of the protein bound state, especially when calculating affinities to very flexible and malleable proteins such as Cythochrome P450s (CYPs) [11,12].

CYPs are able to metabolize a large variety of substrates. This ability can be attributed to the considerable flexibility and plasticity of their active site. For example, work of Ekroos and Sjögren [13] showed that upon substrate binding, CYP 3A4 can substantially alter its conformation and it can increase the size of its active site by more than 80%. Also another drug metabolizing CYP, 2D6, is known to be able to substantially adapt its conformation upon substrate binding, depending on the size and properties of the substrate [14,15]. These examples are not unique in this family of enzymes, and as it is suggested in a review by Pochapsky *et al.* [16], CYP enzyme structure must be considered in four dimensions, as flexible and dynamic arrangements.

Proteins with large and flexible active sites can accommodate ligands in various orientations. Hence, the choice of the initial binding mode used to start MD simulations from is crucial for the parametrization and accuracy of the designed LIE model. To tackle this problem, we recently proposed an iterative scheme that makes it possible to include multiple independent simulations (starting from different ligand-binding modes) into a single LIE model. Using a Boltzmann-weighting approach, the relative contributions of the various simulations to the binding free energy are automatically obtained [17]. Inclusion of different ligand poses was found to significantly increase the accuracy of a LIE model for CYP 2C9 binding, in line with recent theoretical studies of Faver *et al.* [18] highlighting the importance of local sampling to reduce errors in free energy computation. The obtained increase in accuracy showed the advantage of the iterative approach, especially in cases when binding modes have not been experimentally determined.

For flexible proteins, developing accurate LIE models is additionally challenged by difficulties in covering the protein-conformational space during MD simulations of the protein-ligand complex. Evidently, the large number of conformations available to CYPs or other flexible proteins makes full sampling and enumeration of available protein-conformational space problematic. As a remedy, we propose to extend the iterative LIE scheme to also include and combine results from multiple MD simulations starting from different protein conformations. Here, we illustrate the strength of this approach by developing LIE models for CYP 2D6, which is known for its large active-site malleability and for which distinct catalytically-active conformations were recently identified by Hritz *et al.* [14].

Cytochrome P450 2D6 accounts for only a few percent of all human CYPs but it metabolizes more than 20% of the clinical drugs on the current market [19,20]. The first crystal structure of CYP 2D6 (PDB code 2F9Q, 2006, Rowland *et al.* [21]) revealed an active-site cavity with an estimated volume of approximately 0.54 nm^3^, which is larger than for CYPs 2A6, 2A13, 1A2 and 2E1, but smaller than for 2R1, 3A4, 2C8, and 2C9. Hritz *et al.* studied the impact of the plasticity and flexibility of CYP 2D6 on the accuracy of docking-based Site-of-Metabolism (SOM) prediction [14] and revealed that not only large conformational changes but also thermal motion can influence the reliability of structure-based SOM prediction. Interestingly, it was possible to select from MD simulations only a few CYP 2D6 structures that can be used as docking templates to obtain SOM prediction accuracies of more than 80% [14]. Later, positional fluctuations observed during MD [14] of side chains of active-site residues (Glu216, Phe483) could be confirmed by comparison to newly published X-ray structures (PDB codes 3QM4 [15], 3TDA and 3TBG) [22].

**Figure 1.**
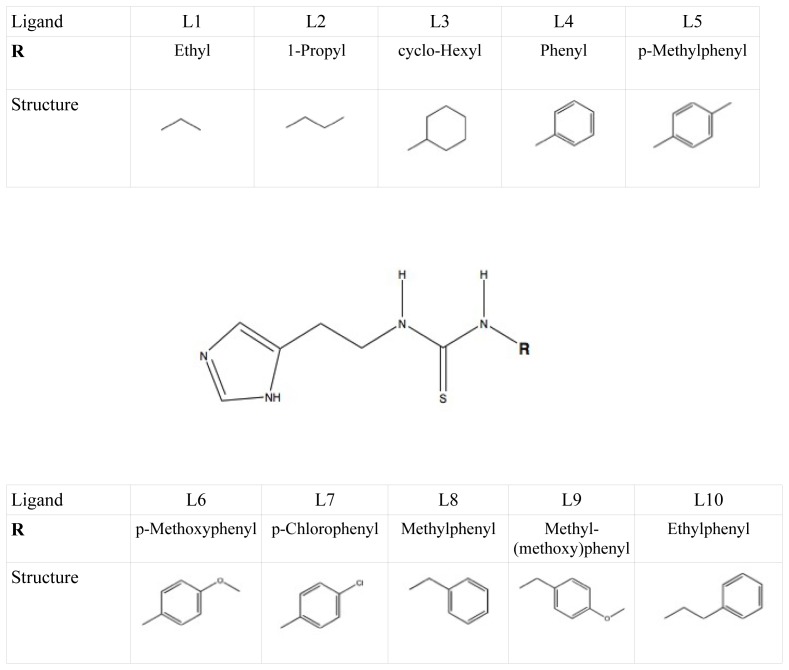
Set of thiourea-containing compounds used to develop a CYP 2D6 LIE model for.

Here, we use the structures of Hritz *et al.* as template coordinate sets to start different MD simulations of substrate-bound CYP 2D6. We will demonstrate the importance of selecting distinct protein conformations (additional to diverse ligand orientations) for the construction of an accurate LIE model, by developing a CYP 2D6 LIE model for thiourea compounds (Table 1 and Figure 1), for which relevant binding orientations could be selected manually. In a follow-up study [23], we show how our iterative LIE method can be used for automated binding affinity prediction for a series of CYP 2D6 binders with a large diversity of possible binding modes, for which *a priori* selection of relevant binding modes (based on visual inspection) is not possible.

## 2. Iterative LIE approach

According to the LIE method [9], free energies of ligand binding are estimated from ensemble-averaged electrostatic $\langle V_{lig-surr}^{EL}\rangle$ and van der Waals interaction energies $\langle V_{lig-surr}^{VdW}\rangle$ between the ligand and its surrounding in the protein-bound (ligand in protein) and in the free state (ligand free in water), as obtained from separate MD simulations. The binding free energy Δ*G_i_* for the ligand binding in pose *i* to the protein is then calculated as

(1) $$\Delta G^{i}=\beta ({\langle V_{lig-surr}^{EL}\rangle }_{protein,i}-{\langle V_{lig-surr}^{EL}\rangle }_{free})+\alpha ({\langle V_{lig-surr}^{VdW}\rangle }_{protein,i}-{\langle V_{lig-surr}^{VdW}\rangle }_{free})$$

*α* and *β* are empirical parameters for the van derWaals and electrostatic contribution of the nonbonded interaction energy to the free energy of binding, respectively. *β* was originally set to 0.5 (according to linear response theory) and only *α* was calibrated using sets of training compounds with experimentally known binding free energies [9]. Later it was shown that coefficient *β* can vary from its theoretical value [10]. Here, *β* is trained in the LIE parameterization procedure as well.

To account for the flexibility of the protein-ligand complex, ${\langle V_{lig-surr}^{EL}\rangle }_{protein}$ and ${\langle V_{lig-surr}^{VdW}\rangle }_{protein}$ are in the current study obtained by performing for every ligand a series of (short) MD simulations of the protein-bound state, with each simulation starting from a different protein-ligand starting structure.

The relative contribution *W_i_* of an independent simulation *i* to the interaction energy of the protein-bound ligand with its surrounding can be calculated [17] as

(2) $$W_{i}=\frac{e^{\frac{-\Delta G^{i}}{k_{B}T}}}{\sum_{i}e^{\frac{-\Delta G^{i}}{k_{B}T}}}$$

such that the binding free energy Δ*G_calc_* of that ligand averaged over the *i* independent simulations can be calculated from

(3) $$\Delta G_{calc}=\beta \sum_{i}W_{i}({\langle V_{lig-surr}^{EL}\rangle }_{protein,i}-{\langle V_{lig-surr}^{EL}\rangle }_{free})+\alpha \sum_{i}W_{i}({\langle V_{lig-surr}^{VdW}\rangle }_{protein,i}-{\langle V_{lig-surr}^{VdW}\rangle }_{free})$$

The W*_i_*’s are obtained by applying an iterative scheme, as described by Stjernschantz and Oostenbrink [17]. After an initial guess, the LIE coefficients *α* and *β* are iteratively optimized until convergence is reached, *i.e.*, by obtaining a minimum value for the root-mean-square error between calculated and experimental values for the binding free energies. Relative experimental binding free energies Δ*G_exp_* for the considered thioureas were derived from inhibition data reported by Onderwater [24] (Table 1).

## 3. Results and Discussion

When calibrating LIE models using results from a single simulation per starting conformation (*i.e.*, using either the S1 or S2 subset of MD simulations, Section 4.3), different values were obtained for LIE parameters and for the root-mean-square errors (RMSEs) between calculated (Δ*G_calc_*) and experimental binding free energies (Δ*G_exp_*). Tables 2 and 3 report *α*, *β* and RMSE values for the different LIE models as obtained for the S1 and S2 sets of simulations, together with errors in the prediction for the compounds (outliers) for which Δ*G_calc_* deviates by more than 1 kcal mol*^−^*^1 (^4.184 kJ mol*^−^*^1^) from Δ*G_exp_*.

The first four models in Tables 2 and 3 (designated as P70-M1, P70-M2, P170-M1, and P170-M2) correspond to traditional LIE models and include per ligand results of a single MD simulation of the protein-bound state, starting from binding pose M1 (Section 4.2) in the P70 conformation (Section 4.1) in case of P70-M1, *etc*. The P70 and P170 models in Tables 2 and 3 combine results of the two simulations starting from either the M1 or M2 orientation in the corresponding protein structure, whereas the M1 and M2 models are based on the sets of two simulations starting from the M1 or M2 pose, respectively, in either of the two protein conformations. Finally, “all” in Tables 2 and 3 refers to the LIE models that include results from simulations starting from any of the four starting configurations.

From the results presented in Tables 2 and 3, one may conclude that inclusion of different starting configurations in the calculations does not necessarily improve the correlation between experimental and calculated values for the binding free energy. For example, when considering the S1 subset of simulations (Table 2), the lowest RMSE value and number of outliers was obtained for the P70-M2 model, which was calibrated from single protein-ligand simulations per compound. However, the S2 subset of simulations indicates that the lowest RMSE was obtained for the M1 model instead, whereas the P70-M2 model shows the highest number of outliers of the S2-based models (Table 3). These discrepancies suggest that interaction energies are not converged in a single MD simulation starting from a given protein-ligand conformation. This is also illustrated by the finding that from the S1 and S2 models based on one simulation per ligand, the lowest RMSE was obtained when starting MD from the P70-M2 and the P70-M1 conformation, respectively, whereas in the S1 and S2 models that combine results from all four MD runs, the simulations starting from the P70-M2 and P70-M1 conformations showed the largest contribution to Δ*G_calc_* for only one (S1) or five (S2) of the ligands (see Supplementary Material, Table S1).

**Figure 2.**
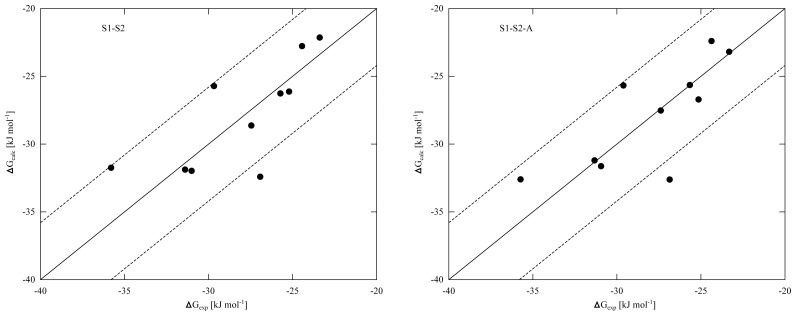
Correlation between calculated (Δ*G_calc_*) and experimental (Δ*G_exp_*) binding free energies obtained for LIE models S1–S2 (**a**) and S1-S2-A (**b**), which combine results from all MD simulations. The solid line indicates ideal correlation between Δ*G_exp_* and Δ*G_calc_*, and thin dashed lines represent errors of *±*4.184 kJ mol*^−^*^1^ (1 kcal mol*^−^*^1^).

**Figure 3.**
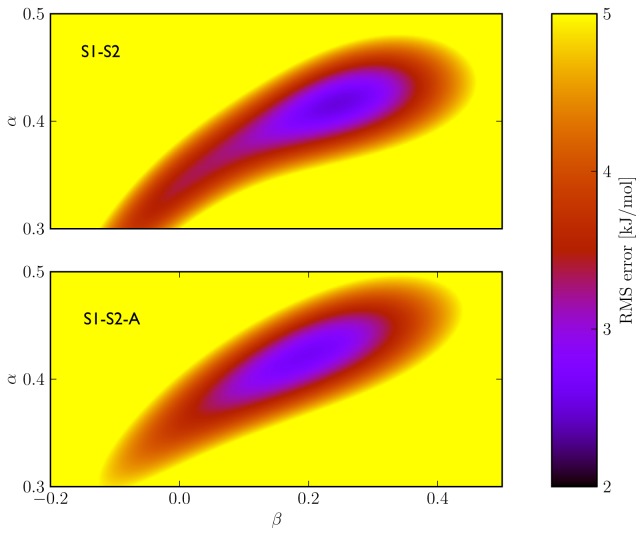
3D contour plots of the dependency of the RMSE (root-mean square error between calculated and experimental binding free energies) on *α* and *β* values for the S1–S2 (**a**) and S1-S2-A (**b**) LIE models, which are based on inclusion of results from all MD simulations. The color bar indicates a range of RMSE values between 2 and 5 kJ mol*^−^*^1^. RMSE values of 5 kJ mol*^−^*^1^ and higher are depicted in yellow.

To improve convergence of average ligand-protein interaction energies, results from the S1 and S2 simulation sets were combined to calibrate the LIE models presented in Tables 4 and 5. Combination of the S1 and S2 results can be achieved in two ways. The first combination scheme was used in calibrating the LIE models in Table 4 (referred to as S1–S2) and considers every S1 and S2 simulation separately in Equation (3), also S1 and S2 runs that start from the same protein-thiourea configuration. As pointed out in reference [17], use of Equation (3) to calculate Δ*G_bind_* from multiple MD simulations per ligand requires that individual simulations cover different parts of protein-ligand conformational space. For several ligands, different hydrogen bond interactions with the protein were observed when comparing pairs of S1 and S2 simulations starting from the same protein-ligand conformation (see Supplementary Material, Table S2 and Figure S1). This can be seen as a motivation to treat S1 and S2 simulations individually, as in the calibration of the S1–S2 models. On the other hand, relatively short MD simulations were performed in this study, justified by the fact that conformational sampling is already partly achieved by including results from simulations starting from different protein conformations and ligand-binding orientations. Therefore, large conformational changes cannot be expected during a single simulation and based on this argument, pairs of S1 and S2 simulations starting from a single configuration should not be assigned separate weights *W_i_* in calibrating the LIE models. In the S1-S2-A models presented in Table 5, 〈*V_EL_*〉 and 〈*V_V dW_*〉 obtained from pairs of S1 and S2 simulations (starting from the same protein-ligand conformation) were averaged for use in Equation (3). Note that in the limit of infinite sampling, the ensemble averages of the interaction energies would be identical for simulations S1 and S2, leading to identical weights (Equation 2) and the difference between models S1-S2 and S1-S2-A disappears. From the RMSEs of the S1-S2 and S1-S2-A models (Tables 4 and 5) and the obtained correlation between calculated and experimental binding affinities (Figure 2), we found similar performance in binding free energy prediction when using either one of the schemes, despite of the slight differences in calibrated *β* values (Tables 4 and 5). In addition, the S1–S2 and S1-S2-A sets demonstrate a similar profile in terms of the dependence of the model’s RMSE on the calibrated *α* and *β* values (Figure 3), which also shows a larger sensitivity of the RMSE on the *α* than on the *β* parameter. This indicates a larger dependency of the predicted binding free energy on differences in (apolar) van der Waals interactions than on changes in electrostatic interactions between ligand and environment upon binding, in line with an earlier LIE model for CYP 1A2 as presented by Vasanthanathan *et al.* [25]. For CYP 1A2, Vasanthanathan found that the predictive accuracy of the free-energy models improved upon forcing *β* to zero in Equation (1) and introducing a constant *γ* as additional off-set parameter. Here, we found for some of the models in Tables 2–5 a small decrease in RMSE when recalibrating our models using *α* and *γ* as fitting parameters (instead of *α* and *β*). For example, for the models in Tables 2–5 in which *β* was found to adopt an unphysical (negative) value, RMSEs were found to decrease by 0.15 (M1/S1), 0.66 (P170/S2), 0.03 (P170/S1-S2) and 0.14 kJ mol*^−^*^1^ (P170/S1-S2-A), respectively. However, when fitting a model with *α* and *γ* as parameters and using results from *all* simulations, we found an increase in RMSE by 0.62 kJ mol*^−^*^1^ (S1–S2) and 0.72 kJ mol*^−^*^1^ (S1-S2-A) when compared to values in Tables 4 and 5, indicating that the dependency of the binding free energy on electrostatic interactions should not be neglected in these cases.

When improving MD sampling by combining results from the S1 and S2 subsets of simulations using the S1–S2 scheme, the LIE model with lowest RMSE from experiment and lowest number of outliers (1) was obtained when combining results from all simulations (Table 4). Also when using the S1-S2-A scheme, the LIE model that includes results from all simulations shows a lower RMSE and fewer outliers than the models that were calibrated based on smaller sets of simulations. Only the S1-S2-A M2 model shows a slightly lower RMSE than the S1-S2-A ‘all’ model (by 0.26 kJ mol*^−^*^1^, Table 5), but when including simulations starting from the M1 poses as well, a *β* value was obtained that is in turn slightly closer to the theoretical value of 0.5 [9]. Moreover, when inspecting contributions from the sets of simulations included in the S1-S2 and S1-S2-A LIE models based on all MD runs (Tables 6 and 7), significant contributions to the predicted free energies were not only obtained from simulations starting from the M2 pose, but also from those starting from M1 (with the latter even dominating for most of the ligands in case of S1-S2-A, Table 7).

The finding that different thiourea binding orientations contribute to predicted CYP 2D6 binding affinities is in line with our earlier work [17], in which significant contributions from multiple ligand-binding modes on the accuracy of predicting CYP 2C9 binding free energies were observed. To develop an accurate LIE model for CYP 2C9, it was found sufficient to combine results from different simulations using the same protein conformation to start MD from [17]. Here we show that for the very flexible CYP 2D6 enzyme, predictions improve upon including simulations starting from different protein conformations, as illustrated in Tables 4 and 5 by the decreased RMSE when comparing LIE models based on all simulations with the P70 and P170 models. It should be noted that especially for the S1-S2-A P70 model, this decrease in RMSE is relatively small (0.19 kJ mol*^−^*^1^, Table 5). On the other hand, when comparing the LIE models based on all simulations with the P170 models, improvement is also observed in terms of a significant increase in *β*. In addition, both protein conformations contribute significantly to the predicted binding free energies (Tables 6 and 7). These findings demonstrate the importance for the flexible Cytochrome P450 2D6 enzyme of including MD simulations starting from different protein-ligand conformations to obtain accurate binding affinities. Moreover, because sampling of protein-ligand conformational space is already partly accounted for by combining MD simulations starting from distinct configurations, relatively short MD simulations (1 ns) are sufficient to obtain RMSE errors well below 1 kcal mol*^−^*^1^. In contrast, calculation of relative binding free energy differences between any pair of ligands by using rigorous free energy methods such as thermodynamic integration [8] or free-energy perturbation [7] would probably require a series of simulations on the nanosecond time scale for every perturbation of a given ligand into another one. In summary, the chosen approach does not only improve the predictive quality of the method, but also its computational attractability and efficiency. Because our approach relies on averaging over multiple independent simulations, its efficiency can be optimized by using implementations of MD software on massively parallel computing facilities that are available within e.g., the Folding@Home [26] andWeNMR communities [27]. An additional strength of the chosen approach is that *a priori*, no knowledge is required of the dominant protein configuration. In a follow-up study [23] we will show how our approach can be automated (e.g., for industrial application) by developing a LIE model for a different class of CYP 2D6 binders, for which pre-selection of possible ligand-binding modes (based on visual inspection of docked ligand poses) is not feasible.

## 4. Computational Methods

### 4.1. Choice of the Protein Coordinate Templates

By docking 65 CYP 2D6 substrates with known SOM into a set of 2500 protein structures obtained from MD simulations, Hritz *et al.* could select three CYP 2D6 conformations as templates for docking-based SOM predictions to reach an accuracy of up to 80% [14]. Following a simple binary decision tree based on the substrate’s molecular weight and number of hydrophobic atoms, it is decided which of the template structures should be used to dock a given ligand into. Hritz *et al.* could characterize the structural diversity between the CYP 2D6 docking templates in terms of the rotameric state of the bulky Phe483 residue in a flexible loop within the active site. To obtain maximum accuracy in SOM prediction using two 2D6 template structures only, it was found that small substrates should be docked into a conformation with the dihedral angle *χ*_1_ of Phe483 adopting a value of approximately 70*°*, whereas the docking template used for large compounds exhibits a *χ*_1_ value of 170*°*. To account for this structural variety, we started our docking and LIE/MD studies from two CYP 2D6 structures that were identified by Hritz to give maximum accuracy in docking-based SOM predictions, and which differ in the Phe483 *χ*_1_ values. In the current work, P70 (*χ*_1_ ≅ 70*°*) refers to the first of these structures (denoted as PPD-70-fr-216 in the Hritz paper), and the second one (referred to as PPD-170-fr-173 in the Hritz paper) is denoted here as P170 (*χ*_1_ ≅ 170*°*).

### 4.2. Docking

**Figure 4.**
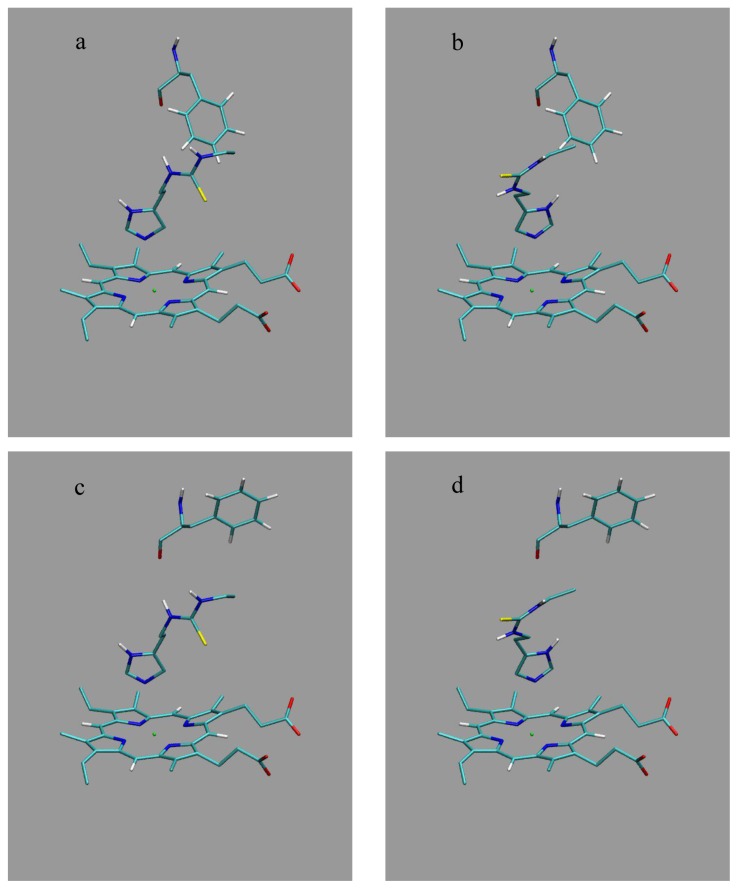
Typical ligand-binding orientations to start MD simulations from: (**a**) pose P70-M1; (**b**) pose P70-M2; (**c**) pose P170-M1; and (**d**) pose P170-M2. Residue Phe483, ligand (L1) and the CYP heme group are shown.

Docking of the thiourea compounds into the active site of the two CYP 2D6 protein structures was performed by using GOLD (Genetic Optimization for Ligand Docking [28]) version 4.0, together with the Chemscore scoring function [29] that was parameterized for heme-containing proteins [30]. For every combination of ligand (using one starting conformation for every ligand) and protein template, 50 docking runs were performed with maximally 1000 genetic algorithm operations using populations of 100 genes. The center point of ligand docking was placed within 0.01 nm of the heme iron atom, using a docking radius of 1.5 nm. As shown in a recent study of Santos *et al.* [31], inclusion of water molecules in the active site during docking showed a strong dependence on the protein conformation used, and no statistically significant improvement of the prediction of catalytically active binding orientations upon inclusion of water molecules in the active site was observed. Therefore, water molecules were not taken into account during the docking procedure. The 50 highest scored binding poses were visually inspected. In every protein template structure, two different ligand-binding poses were selected according to the following criteria: (*i*) the proximity of the ligand imidazole-N*_ε_* atom toward the heme iron (which was chosen to be maximally 0.6 nm [32]) and (*ii*) the orientation of the imidazole ring with respect to the heme group (Figure 4). In the first of the selected poses (denoted as M1), the imidazole-C*_δ_* atom of the ligand is directed towards the heme carboxyl groups (see e.g., Figure 4a,c). In the second selected pose (denoted as M2), C*_δ_* is directed in the opposite direction (Figure 4b,d). By combining the two different protein structures (P70 in Figure 4a,b and P170 in Figure 4c,d) and ligand binding modes (M1 and M2), four different protein-ligand complex conformations were chosen per ligand to start MD simulations from.

### 4.3. Molecular Dynamics Simulation

All MD simulations were performed using the GROMOS11 program [33], together with the GROMOS 45A4 force field [34] and ligand force field parameters described in reference [17]. A total of 90 explicit-solvent NPT simulations were carried out involving the ligand bound to the protein (80 simulations) or the ligand free in water (10 simulations), at a temperature of 300 K and a pressure of 1 atm. The simulations were carried out under periodic boundary conditions, after solvating energy-minimized molecular structures in pre-equilibrated rectangular computational boxes filled with SPC water molecules [35]. The box length was set to 8.3 nm (ligand in protein) and 3.3 nm (ligand in water), and molecular structures were solvated by *~*16,400 or *~*1200 water molecules, respectively. Six water molecules were replaced by Na^+^ counter ions (ligand in protein), in order to neutralize the net charge of the protein-ligand complex, after which the system was energy minimized again. During MD simulations, the equations of motion were integrated using the leap-frog scheme [36] with a timestep of 2 fs. The SHAKE algorithm [37] was applied to constrain all bond lengths to their zero-energy value, using a relative geometric tolerance of 10*^−^*^4^. The value of the *χ*_1_ dihedral angle of protein residue Phe483 was restrained to either 70*°* or 170*°* using a harmonic potential with a force constant of 30.0 kJ mol*^−^*^1^deg*^−^*^2^, in order to prevent possible transitions between P70 and P170 protein conformations during simulation and to ensure that different parts of protein-ligand conformational space were sampled in independent MD simulations (which is a prerequisite for use of Equation (2) to calculate their relative weights [17]). During MD, the temperature was maintained close to its reference value (300 K) by weakly coupling the solute and solvent degrees of freedom separately to a heat bath [38], with a relaxation time of 0.1 ps. The pressure was maintained close to its reference value (1 atm) by weak coupling of the particle coordinates and box dimensions (isotropic coordinate scaling) to a pressure bath [38], using a relaxation time of 0.5 ps and an isothermal compressibility of 0.4575 .10*^−^*^3^ kJ*^−^*^1^ mol nm^3^. Non-bonded interactions were computed using a twin-range scheme [39,40], with short- and long-range cutoff distances set to 0.8 and 1.4 nm, respectively, and a frequency of 5 time steps for the update of the short-range pairlist and for the calculation of intermediate-range interactions. A reaction-field correction [41] was applied to account for the mean effect of omitted electrostatic interactions beyond the long-range cutoff distance, using a relative dielectric permittivity of 61, which is appropriate for the SPC water model [42]. During the first 100 ps of equilibration, the temperature was increased in a stepwise manner up to 300 K, by increasing the temperature by 60 K every 20 ps and by applying harmonic positional restraints to the protein backbone atoms, using a force constant decreasing from 25,000 kJ mol*^−^*^1^ nm*^−^*^1^ (at 60 K) to 25 kJ mol*^−^*^1^ nm*^−^*^1 (^at 240 K). After an equilibration period of in total 2.1 ns, all simulations were carried out for a duration of 1 ns during which interaction energies and coordinates were stored every 0.02 and 0.4 ps, respectively, for subsequent analysis. Starting from all (four) combinations of protein conformations (P70 and P170) and ligand-binding orientations (M1 and M2), two independent MD simulations were performed (designated as S1 and S2, respectively). For each pair of S1 and S2 simulations, the same starting conformation of the protein-thiourea complex was used, but different (random) sets of atomic velocities were assigned at the beginning of the thermal equilibration phase.

## 5. Conclusions

In this study, we used an iterative LIE method to develop a MD-based model for the prediction of binding free energies of a series of thioureas to CYP 2D6. A Boltzmann-weighting LIE scheme as recently introduced by Stjernschantz and Oostenbrink [17] was enriched by including MD simulations starting from different protein conformations for every ligand. Here, we show that this inclusion improves ligand-protein affinity predictions for the very flexible CYP 2D6 isoform. It is also shown that, starting from a specific ligand-protein conformation, a single ns-scale MD simulation is not sufficient to obtain converged protein-ligand interaction energies. This convergence issue can be resolved by combining sets of relatively short MD simulations starting from an identical conformation, either as independent simulations, or by averaging their electrostatic and van der Waals interaction energies before applying the Boltzmann-weighting scheme. Use of both averaging schemes led to excellent agreement with experimental data when combining results from MD simulations starting from four different protein-ligand conformations, which resulted in a LIE model with RMSE (root-mean-square error between predicted and experimental binding free energies) well below 1 kcal mol*^−^*^1^. The applied LIE approach serves as a promising template to develop efficient ligand-binding affinity prediction tools for very flexible and malleable proteins such as CYPs.

## Supplementary file



## Figures and Tables

**Table 1.** Experimental binding free energies Δ*G_exp_* for the thiourea compounds considered, derived from *IC*_50_ values reported in a CYP 2D6 inhibition study with 7-methoxy-4(aminomethyl)coumarin (MAMC) as a fluorometric probe [24].

LIGAND	R	*IC*_50_(*μ*M)	Δ*G_exp_*(kJ mol^−1^)
L1	Ethyl	87 *±* 21	*−*23.32 *±* 0.69
L2	1-Propyl	42 *±* 14	*−*25.14 *±* 1.01
L3	*cyclo*-Hexyl	34 *±* 15	*−*25.66 *±* 1.45
L4	Phenyl	57 ± 17	*−*24.37 ± 0.88
L5	*p*-Methylphenyl	17 ± 6.0	*−*27.39 ± 1.09
L6	*p*-Methoxyphenyl	21 ± 8.7	*−*26.86 ± 1.34
L7	*p*-Chlorophenyl	3.5 ± 1.2	*−*31.33 ± 1.05
L8	Methylphenyl	7.0 ± 1.2	*−*29.61 ± 0.47
L9	Methyl-(*p*-methoxy)phenyl	4.1 ± 1.4	*−*30.94 ± 1.04
L10	Ethylphenyl	0.60 ± 0.13	*−*35.73 ± 0.61

**Table 2.** *α* and *β* parameters for the LIE models obtained from simulations S1. For each model, root-mean-square errors (RMSEs, in kJ mol*^−^*^1^) between calculated (Δ*G_calc_*) and experimental binding free energies (Δ*G_exp_*) are shown as well, together with errors (in kJ mol*^−^*^1^) in the prediction for the compounds (outliers) for which Δ*G_calc_* deviates by more than 1 kcal mol*^−^*^1^ (4.184 kJ mol*^−^*^1^ ) from Δ*G_exp_*.

Runs	MODEL	*β*	*α*	RMSE	L1	L2	L3	L4	L5	L6	L7	L8	L9	L10
S1	P70-M1	0.239	0.425	3.65	5.01					7.69				4.77
	P70-M2	0.142	0.434	1.39										
	P170-M1	0.084	0.414	4.07				5.46				5.18	6.78	
	P170-M2	0.231	0.493	4.03			6.43				6.39			6.45
	P70	0.223	0.416	2.95						7.03		4.26		
	P170	0.066	0.398	3.68				4.24			4.98	4.61	5.30	4.33
	M1	−0.142	0.301	3.17				6.69						6.17
	M2	0.174	0.437	1.40										

	all	0.219	0.415	2.78						6.4		4.82		

**Table 3.** *α* and *β* parameters for the LIE models obtained from simulations S2. For each model, root-mean-square errors (RMSEs, in kJ mol*^−^*^1^) between calculated (Δ*G_calc_*) and experimental binding free energies (Δ*G_exp_*) are shown as well, together with errors (in kJ mol*^−^*^1^) in the prediction for the compounds (outliers) for which Δ*G_calc_* deviates by more than 1 kcal mol*^−^*^1^ (4.184 kJ mol*^−^*^1^ ) from Δ*G_exp_*.

Runs	MODEL	*β*	*α*	RMSE	L1	L2	L3	L4	L5	L6	L7	L8	L9	L10
S2	P70-M1	0.153	0.400	2.70						4.19				5.50
	P70-M2	0.119	0.405	4.61	4.20	6.33				8.49		6.78		5.42
	P170-M1	0.048	0.407	4.26										11.35
	P170-M2	0.031	0.412	5.56						7.11	11.10			10.65
	P70	0.176	0.404	3.38						6.32				5.73
	P170	−0.069	0.351	4.28				4.52						11.10
	M1	0.116	0.392	2.60										5.29
	M2	0.107	0.395	3.80		5.65				7.17				6.39

	all	0.146	0.397	3.18						5.70				5.98

**Table 4.** *α* and *β* parameters for the LIE models obtained from simulation sets S1 and S2, by considering S1 and S2 sets separately in Equation (3). For each LIE model, root-mean-square errors (RMSEs, in kJ mol*^−^*^1^) between calculated (Δ*G_calc_*) and experimental binding free energies (Δ*G_exp_*) are shown as well, together with errors (in kJ mol*^−^*^1^) in the prediction for the compounds (outliers) for which Δ*G_calc_* deviates by more than 1 kcal mol*^−^*^1^ (4.184 kJ mol*^−^*^1^) from Δ*G_exp_*.

Runs	MODEL	*β*	*α*	RMSE	L1	L2	L3	L4	L5	L6	L7	L8	L9	L10
S1–S2	P70-M1	0.109	0.381	3.15						5.24				6.47
	P70-M2	0.120	0.400	3.70		5.45				7.66				4.53
	P170-M1	0.030	0.381	3.19				4.25			4.84		4.79	4.61
	P170-M2	0.010	0.394	4.79						5.33	7.12			10.72
	P70	0.215	0.411	2.92						6.37		4.30		
	P170	*−*0.056	0.343	3.11							4.24			5.93
	M1	0.063	0.366	3.02										6.49
	M2	0.162	0.406	3.22						6.63		4.26		6.39

	all	0.217	0.411	2.55						5.75				

**Table 5.** *α* and *β* parameters for the LIE models obtained from simulation sets S1 and S2, by averaging results for pairs of S1 and S2 simulations. For each LIE model, root-mean-square errors (RMSEs, in kJ mol*^−^*^1^) between calculated (Δ*G_calc_*) and experimental binding free energies (Δ*G_exp_*) are shown as well, together with errors (in kJ mol*^−^*^1^) in the prediction for the compounds (outliers) for which Δ*G_calc_* deviates by more than 1 kcal mol*^−^*^1^ (4.184 kJ mol^−1^) from Δ*G_exp_*.

Runs	MODEL	*β*	*α*	RMSE	L1	L2	L3	L4	L5	L6	L7	L8	L9	L10
S1-S2-A	P70-M1	0.146	0.401	3.09						5.78				6.11
	P70-M2	0.149	0.428	2.78						5.62				
	P170-M1	0.017	0.400	3.34				5.39					4.53	6.75
	P170-M2	0.142	0.455	4.58						4.42	7.30			10.18
	P70	0.203	0.419	2.78						5.90				
	P170	−0.000	0.389	3.45				4.54						7.87
	M1	0.108	0.395	3.13						4.97				6.89
	M2	0.170	0.430	2.33						4.29				

	all	0.190	0.419	2.59						5.50				

**Table 6.** Relative weights *W_i_* of the different simulations *i* to the binding free energies calculated for ligands L1–L10 using the S1–S2 LIE model (as obtained by combining results from all simulations).

Ligand	P70-M1-S1	P70-M2-S1	P170-M1-S1	P170-M2-S1	P70-M1-S2	P70-M2-S2	P170-M1-S2	P170-M2-S2
L1	0.026	0.165	0.218	0.063	0.101	0.011	0.030	**0.386**
L2	0.091	0.047	0.029	0.018	0.120	**0.630**	0.053	0.013
L3	**0.366**	0.004	0.059	0.257	0.213	0.028	0.025	0.047
L4	0.157	0.014	**0.392**	0.145	0.113	0.034	0.113	0.031
L5	**0.371**	0.025	0.057	0.075	0.166	0.014	0.286	0.006
L6	**0.268**	0.004	0.118	0.001	0.113	0.261	0.116	0.119
L7	0.225	0.168	0.022	0.003	0.185	**0.391**	0.002	0.005
L8	0.150	0.113	0.008	0.027	0.206	0.046	**0.309**	0.140
L9	0.078	0.019	**0.313**	0.024	0.199	0.060	0.064	0.243
L10	0.098	**0.621**	0.056	0.010	0.074	0.136	0.003	0.001

**Table 7.** Relative weights *W_i_* of the different simulations *i* to the binding free energies calculated for ligands L1-L10 using the S1-S2-A LIE model (as obtained by combining results from all simulations).

Ligand	P70-M1	P70-M2	P170-M1	P170-M2
L1	0.177	0.150	0.266	**0.408**
L2	0.309	**0.538**	0.108	0.044
L3	**0.608**	0.038	0.086	0.267
L4	0.293	0.068	**0.483**	0.156
L5	**0.663**	0.063	0.219	0.055
L6	**0.556**	0.137	0.264	0.043
L7	**0.515**	0.461	0.017	0.007
L8	**0.473**	0.189	0.146	0.192
L9	0.341	0.085	**0.387**	0.187
L10	0.257	**0.687**	0.047	0.009

## References

[1] Christ C.D., Mark A.E., van Gunsteren W.F. (2010). Basic ingedients of free energy calculations: A review. J. Comp. Chem.

[2] De Ruiter A., Oostenbrink C. (2011). Free energy calculations of protein-ligand interactions. Curr. Opin. Chem. Biol.

[3] Ferrara P., Gohlke H., Price D.J., Klebe G., Brooks C.L. (2004). Assessing scoring functions for protein-ligand interactions. J. Med. Chem.

[4] Warren G.L., Andrews C.W., Capelli A.M., Clarke B., LaLonde J., Lambert M.H., Lindvall M., Nevins N., Semus S.F., Senger S. (2005). A critical assessment of docking programs and scoring functions. J. Med. Chem.

[5] Stjernschantz E., Marelius J., Medina C., Jacobsson M., Vermeulen N.P.E., Oostenbrink C. (2006). Are automated molecular dynamics simulations and binding free energy calculations realistic tools in lead optimization? An evaluation of the linear interaction energy (LIE) method. J. Chem. Inf. Model.

[6] Cheng T., Li X., Li Y., Liu Z., Wang R. (2009). Comparative assessment of scoring functions on a diverse test set. J. Chem. Inf. Model.

[7] Zwanzig R.W. (1954). High-temperature equation of state by a perturbation method. I. Nonpolar gases. J. Chem. Phys.

[8] Beveridge D.L., DiCapua F.M. (1989). Free-energy via molecular simulation—Applications to chemical and biomolecular systems. Annu. Rev. Biophys. Biophys. Chem.

[9] Åqvist J., Medina C. (1994). A new method for predicting binding affinity in computer-aided drug design. Protein Eng.

[10] Hansson T., Marelius J., Åqvist J. (1998). Ligand binding affinity prediction by linear interaction energy methods. J. Comput.-Aided Mol. Des.

[11] Guengerich F.P. (2006). A malleable catalyst dominates the metabolism of drugs. Proc. Natl. Acad. Sci. USA.

[12] Oostenbrink C., de Ruiter A., Hritz J., Vermeulen N.P.E. (2012). Malleability and versatility of cytochrome P450 active sites studied by molecular simulations. Curr. Drug Metab.

[13] Ekroos M., Sjögren T. (2006). Structural basis for ligand promiscuity in cytochrome P450 3A4. Proc. Natl. Acad. Sci. USA.

[14] Hritz J., de Ruiter A., Oostenbrink C. (2008). Impact of plasticity and flexibility on docking results for cytochrome P450 2D6: A combined approach of molecular dynamics and ligand docking. J. Med. Chem.

[15] Wang A., Savas U., Hsu M.H., Stout C.D., Johnson E.F. (2012). Crystal structure of Human Cytochrome P450 2D6 with Prinomastat Bound. J. Biol. Chem.

[16] Pochapsky T.C., Kazanis S., Dang M. (2010). Conformational plasticity and structure/function relationships in Cytochromes P450. Antioxid. Redox Signal.

[17] Stjernschantz E., Oostenbrink C. (2010). Improved ligand-protein binding affinity predictions using multiple binding modes. Biophys. J.

[18] Faver J.C., Yang W., Merz K.M. (2012). The Effects of computational modeling errors on the estimation of statistical mechanical variables. J. Chem. Theo. Comput.

[19] Williams J.A., Hyland R., Jones B.C., Smith D.A., Hurst S., Goosen T.C., Peterkin V., Koup J.R., Ball S.E. (2004). Drug-drug interactions for UDP-glucuronosyltransferase substrates: A pharmacokinetic explanation for typically observed low exposure (AUC(i)/AUC) ratios. Drug Metab. Dispos.

[20] Bazeley P.S., Prithivi S., Struble C.A., Povinelli R.J., Sem D.S. (2006). Synergistic use of compound properties and docking scores in neural network modeling of CYP2D6 binding: Predicting affinity and conformational sampling. J. Chem. Inf. Model.

[21] Rowland P., Blaney F.E., Smyth M.G., Jones J.J., Leydon V.R., Oxbrow A.K., Lewis C.J., Tennant M.G., Modi S., Eggleston D.S., Chenery R.J., Bridges A.M. (2006). Crystal structure of human cytochrome P450 2D6. J. Biol. Chem.

[22] Oostenbrink C., Kirchmair J. (2013). Structure-based Methods for Predicting the Sites and Products of Metabolism. Drug Metabolism Prediction.

[23] Vosmeer C.R., Pool R., van Stee M.F., Vermeulen N.P.E., Geerke D.P. (2013). Towards automated binding affinity prediction using an iterative linear interaction energy approach. Int. J. Mol. Sci.

[24] Onderwater R. (2005). Molecular Toxicology of Thiourea-containing Compounds. Ph.D. Thesis.

[25] Vasanthanathan P., Olsen L., Jørgensen F.S., Vermeulen N.P.E., Oostenbrink C. (2010). Computational prediction of binding affinity for CYP1A2-ligand complexes using empirical free energy calculations. Drug Metab. Dispos.

[26] Pande V.S., Baker I., Chapman J., Elmer S.P., Khaliq S., Larson S.M., Rhee Y.M., Shirts M.R., Snow C.D., Sorin E.J., Zagrovic B. (2003). Atomistic protein folding simulations on the submillisecond time scale using worldwide distributed computing. Biopolymers.

[27] Van Dijk M., Wassenaar T.A., Bonvin A.M.J.J. (2012). A flexible, grid-enabled web portal for GROMACS molecular dynamics simulations. J. Chem. Theory Comput.

[28] Jones G., Willett P., Glen R.C., Leach A.R., Taylor R.D. (1997). Development and validation of a genetic algorithm for flexible docking. J. Mol. Biol.

[29] Eldridge M.D., Murray C.W., Auton T.R., Paolini G.V., Mee R.P. (1997). Binding affinity prediction; Empirical scoring function; De novo molecular design; Protein-ligand complexes. J. Comput. Aided Mol. Des.

[30] Kirton S.B., Murray C.W., Verdonk M.L., Taylor R.D. (2005). Prediction of binding modes for ligands in the Cytochromes P450 and the other heme-containing proteins. Proteins.

[31] Santos R., Hritz J., Oostenbrink C. (2010). Role of water in molecular docking simulations of cytochrome P450 2D6. J. Chem. Inf. Model.

[32] De Graaf C., Oostenbrink C., Keizers P.H.J., van der Wijst T., Jongejan A., Vermeulen N.P.E. (2006). Catalytic site prediction and virtual screening of Cytochrome P450 2D6 substrates by consideration of water and rescoring in automated docking. J. Med. Chem.

[33] Schmid N., Christ C.D., Christen M., Eichenberger A.P., van Gunsteren W.F. (2012). Architecture, implementation and parallelisation of the GROMOS software for biomolecular simulation. Comp. Phys. Commun.

[34] Lins R.D., Hünenberger P.H. (2005). A new GROMOS force field for hexopyranose-based carbohydrates. J. Comp. Chem.

[35] Berendsen H.J.C., Postma J.P.M., van Gunsteren W.F., Hermans J., Pullman B. (1981). Intermolecular Forces.

[36] Hockney R.W. (1970). The potential calculation and some applications. Methods Comput. Phys.

[37] Ryckaert J.-P., Ciccotti G., Berendsen H.J.C. (1977). Numerical integration of the cartesian equations of motion of a system with constraints: Molecular dynamics of n-alkanes. J. Comput. Phys.

[38] Berendsen H.J.C., Postma J.P.M., van Gunsteren W.F., Di Nola A., Haak J.R. (1984). Molecular-dynamics with coupling to an external bath. J. Chem. Phys.

[39] Van Gunsteren W.F., Berendsen H.J.C. (1990). Computer simulation of molecular dynamics: Methodology, applications and perspectives in chemistry. Angew. Chem. Int. Ed. Engl.

[40] Van Gunsteren W.F., Billeter S.R., Eising A.A., Hünenberger P.H., Krüger P., Mark A.E., Scott W.R.P., Tironi I.G. (1996). Biomolecular Simulation: The GROMOS96 Manual and User Guide.

[41] Tironi I.G., Sperb R., Smith P.E., van Gunsteren W.F. (1995). A generalized reaction field method for molecular dynamics simulations. J. Chem. Phys.

[42] Heinz T.N., van Gunsteren W.F., Hünenberger P.H. (2001). Comparison of four methods to compute the dielectric permittivity of liquids from molecular dynamics simulations. J. Chem. Phys.

