# Supplementary material for: CYP 2D6 Binding Affinity Predictions Using Multiple Ligand and Protein Conformations
Source: Int J Mol Sci. 2013 Dec 17;14(12):24514–30. doi: 10.3390/ijms141224514 (PMC3876125; doi:10.3390/ijms141224514)
Supplement: Supplementary file 1 [file ijms-14-24514-s001.pdf]

Supplementary file

Figure S1. Amino acids involved in protein-ligand hydrogen bonds during MD simulation. Indicated are Leu399, Ala272, Glu183, Gly340, Val275, Phe450, Ser271, Asp268 and Thr342. The heme group and ligand (L1) are displayed in dark blue.

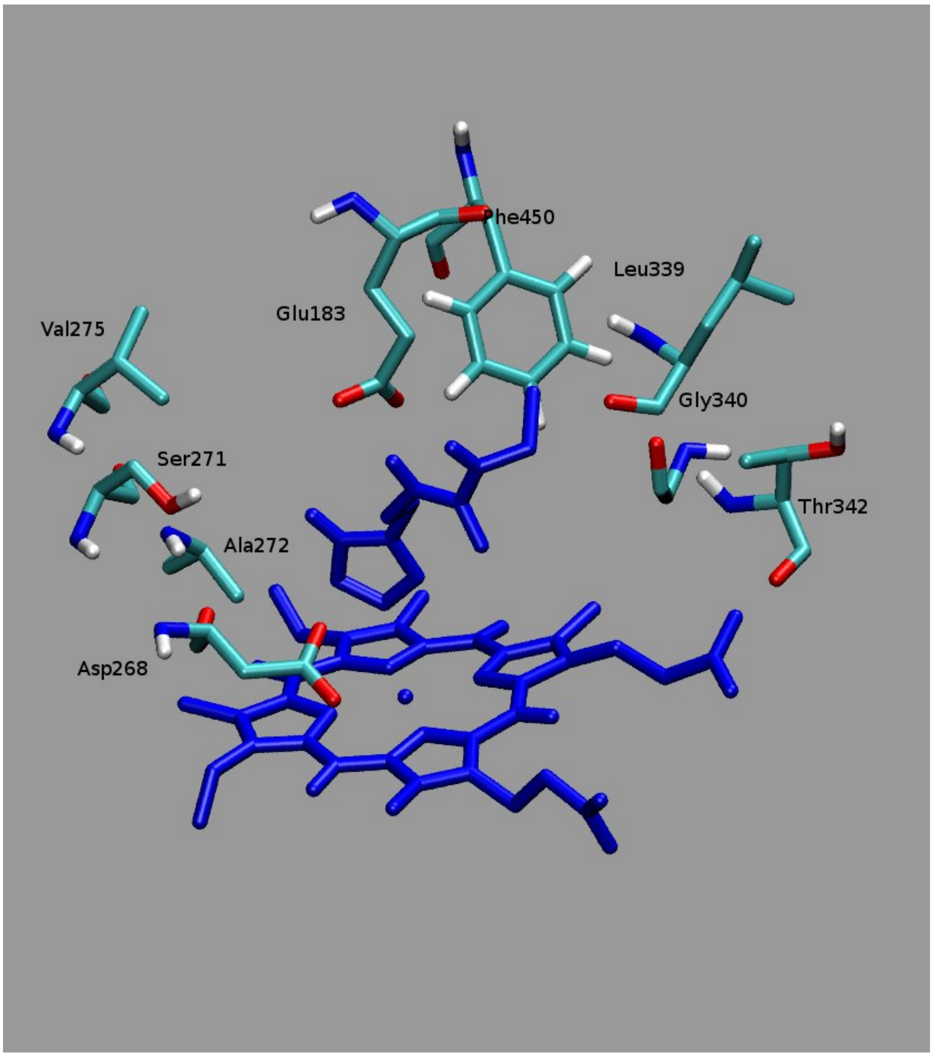

Table S1. Relative weights W_i of the different simulations i to the binding free energies calculated for ligands L1-L10 using the LIE model that was obtained by combining results from all simulations of either the S1 or S2 simulation sets.

Simulation	P70-M1	P70-M2	P170-M1	P170-M2	P70-M1	P70-M2	P170-M1	P170-M2
		**all-S1**				**all-S2**		
L1	0.054	0.351	**0.461**	0.133	0.231	0.050	0.171	**0.548**
L2	**0.493**	0.256	0.156	0.094	0.181	**0.728**	0.074	0.017
L3	**0.535**	0.006	0.085	0.375	**0.541**	0.144	0.129	0.186
L4	0.221	0.020	**0.556**	0.203	0.325	0.175	**0.348**	0.152
L5	**0.707**	0.047	0.105	0.141	**0.583**	0.062	0.336	0.018
L6	**0.690**	0.009	0.298	0.003	0.164	**0.561**	0.081	0.195
L7	**0.543**	0.399	0.052	0.006	0.395	**0.578**	0.012	0.007
L8	**0.503**	0.381	0.025	0.091	**0.395**	0.048	0.328	0.230
L9	0.178	0.042	**0.726**	0.054	**0.599**	0.084	0.097	0.221
L10	0.123	**0.749**	0.071	0.013	**0.536**	0.437	0.020	0.007

Table S2. Occurrence of hydrogen bonds between ligands and CYP 2D6 residues for two sets of simulations (S1 and S2) starting from poses M1-P70, M2-P70, M1-P170 and M2-P170. Occurrence in Set 1 is represented by □ and occurrence in Set 2 is represented by ■ symbols. The occurrence of a hydrogen bond was defined by a maximal acceptor-hydrogen distance of 0.25 nm and a minimal acceptor-hydrogen-donor angle of 135°.

Simulation	Leu399	Ala272	Glu183	Gly340	Val275	Phe450	Ser271	Asp268	Thr342
L1-M1-P70	■ □								
L1-M2-P70		■	□						
L1-M1-P170				□					
L1-M2-P170			■ □						
L2-M1-P70			■	■		□			
L2-M2-P70	□		■						
L2-M1-P170				■					
L2-M2-P170			■						
L3-M1-P70				■					
L3-M2-P70		□	■		■				
L3-M1-P170									
L3-M2-P170	□			□					
L4-M1-P70			□			■			
L4-M2-P70				■					
L4-M1-P170									
L4-M2-P170				■					

Table S2. *Cont.*

Simulation	Leu399	Ala272	Glu183	Gly340	Val275	Phe450	Ser271	Asp268	Thr342
L5-M1-P70			□			■			
L5-M2-P70				■					
L5-M1-P170			□					□	
L5-M2-P170				□					
L6-M1-P70		□		■					
L6-M2-P70			□	■					
L6-M1-P170	□			■ □					
L6-M2-P170									■ □
L7-M1-P70		■		□					
L7-M2-P70			□	■					
L7-M1-P170									
L7-M2-P170			■						■
L8-M1-P70			□						
L8-M2-P70			■ □					■	
L8-M1-P170			□						
L8-M2-P170						□			
L9-M1-P70			□						
L9-M2-P70		■ □							
L9-M1-P170									■
L9-M2-P170			■						□
L10-M1-P70		■		□					
L10-M2-P70	■			□					
L10-M1-P170				□					
L10-M2-P170				■ □					
